# Disseminating evidence-based interventions to new populations: a systematic approach to consider the need for adaptation

**DOI:** 10.1186/1748-5908-10-S1-A49

**Published:** 2015-08-20

**Authors:** Marieke A Hartman, Karien Stronks, Linda Highfield, Stephan W Cremer, Arnoud P Verhoeff, Vera Nierkens

**Affiliations:** 1 University of Texas, School of Public Health, Department of Health Promotion & Behavioral Sciences, Houston, TX 77030, USA; 2 University of Amsterdam, Academic Medical Center, Department of Public Health, Amsterdam, 1105 AZ, The Netherlands; 3 University of Texas, School of Public Health, Department of Management, Policy and Community Health, Houston, TX 77030, USA; 4 Public Health Service of Amsterdam, Department of Epidemiology and Health Promotion, Amsterdam, 1018 WT, The Netherlands; 5 University of Amsterdam, Department of Sociology and Anthropology, Amsterdam, 1012 DK, The Netherlands; 6 Leiden University Medical Center, Department of Public Health and Primary Care, Leiden, 2333 ZD, The Netherlands

## Problem

Audience segmentation or targeting interventions towards population subgroups, such as ethnic or racial groups, is hypothesized to increase the likelihood of intervention's relevancy and effectiveness on promoting health behaviors. Therefore, evidence-based interventions (EBIs) developed for a population other than the one of interest are often considered as non-relevant, hindering dissemination. Adaptation of EBIs to new populations of interest can solve this problem, but is discouraged since it can harm effective elements. There is little guidance on how to critically consider the need for adaptation. We present a framework that guides dissemination and implementation researchers and practitioners on how to make informed decisions on whether or not to adapt interventions or aspects of them.

## Solution

We integrated models of ethnicity-based segmentation/ targeting of interventions, intervention planning, and formative evaluations of complex interventions into one framework to give guidance on how to systematically inform adaptation decisions. First, we suggest needs assessments to inform initial adaptation decisions. The framework distinguishes four intervention aspects to make individual adaptation decisions for (whether to retain aspects or to adapt them): behavioral goals, methods and strategies, intervention execution, and channels for delivery. We argue for assessing differences as well as similarities between target populations to make a subjective evaluation on whether those differences may affect EBIs' effectiveness in a way that they require adaptation. Subsequently, we recommend formative evaluations testing cultural relevancy to revise decisions if necessary, before large-scale implementation and evaluation are done among the new population.

## Conclusion

Our framework can contribute to the dissemination of EBIs to other populations than the ones they were originally developed for. The framework discourages making more adaptations than necessary by critically and systematically assessing the need for adaptation. As a consequence, it increases the likelihood of retaining effective elements.

